# Germ Cells Are Not Required to Establish the Female Pathway in Mouse Fetal Gonads

**DOI:** 10.1371/journal.pone.0047238

**Published:** 2012-10-16

**Authors:** Danielle M. Maatouk, Lindsey Mork, Ashley Hinson, Akio Kobayashi, Andrew P. McMahon, Blanche Capel

**Affiliations:** 1 Department of Cell Biology, Duke University Medical Center, Durham, North Carolina, United States of America; 2 Department of Pediatrics, Division of Hematology-Oncology, Duke University School of Medicine, Durham, North Carolina, United States of America; 3 Department of Stem Cell and Regenerative Biology, Department of Molecular and Cellular Biology and Harvard Stem Cell Institute, Harvard University, Cambridge, Massachusetts, United States of America; McGill University, Canada

## Abstract

The fetal gonad is composed of a mixture of somatic cell lineages and germ cells. The fate of the gonad, male or female, is determined by a population of somatic cells that differentiate into Sertoli or granulosa cells and direct testis or ovary development. It is well established that germ cells are not required for the establishment or maintenance of Sertoli cells or testis cords in the male gonad. However, in the agametic ovary, follicles do not form suggesting that germ cells may influence granulosa cell development. Prior investigations of ovaries in which pre-meiotic germ cells were ablated during fetal life reported no histological changes during stages prior to birth. However, whether granulosa cells underwent normal molecular differentiation was not investigated. In cases where germ cell loss occurred secondary to other mutations, transdifferentiation of granulosa cells towards a Sertoli cell fate was observed, raising questions about whether germ cells play an active role in establishing or maintaining the fate of granulosa cells. We developed a group of molecular markers associated with ovarian development, and show here that the loss of pre-meiotic germ cells does not disrupt the somatic ovarian differentiation program during fetal life, or cause transdifferentiation as defined by expression of Sertoli markers. Since we do not find defects in the ovarian somatic program, the subsequent failure to form follicles at perinatal stages is likely attributable to the absence of germ cells rather than to defects in the somatic cells.

## Introduction

During embryogenesis, sexual differentiation begins with the onset of fetal gonad development. The primordial gonad is bipotential and, in mammals, its fate is normally genetically controlled by the presence or absence of a Y-chromosome, leading to male or female development, respectively. A mixture of somatic cell types and germ cells reside within the primordial gonad. In XY gonads, a subset of somatic cells upregulate *Sry*, the sex-determining gene on the Y-chromosome, leading to differentiation of the supporting cell lineage as preSertoli cells [1], [2]. In the absence of *Sry*, XX supporting cells differentiate as pregranulosa cells [1], [3]. Throughout development, gonadal supporting cells closely interact with germ cells. During fetal life, somatic cells dictate whether germ cells initiate differentiation as spermatogonia or oogonia. However the reciprocal influence of germ cells on somatic cell differentiation before birth is not well understood in mammals.

The influence of germ cells on gonadogenesis is highly variable among vertebrates (reviewed in [4]). In the red-eared slider turtle, *Trachemys scripta*, loss of germ cells does not influence sex determination or morphological differentiation of the testis or ovary before hatching, but whether it affects the eventual differentiation of the adult organs is unknown [5]. In contrast, in some species of fish the earliest sexually dimorphic event occurs not in the somatic cells of the gonad, but in the germ cell lineage (reviewed in [6]). In zebrafish, loss of germ cells invariably leads to the differentiation of a testis [7]. The number of germ cells also controls the fate of the gonad in medaka, where mutants that produce a large number of germ cells are female, and those that deplete germ cells are male [8], [9]. In fact, recent studies showed that the medaka *sox9b* gene, which was thought to control testis development in a manner similar to mammalian *Sox9*, does not affect testis differentiation directly, but instead does so through controlling germ cell proliferation [10]. These studies suggest that in some species, germ cells play an active role in the sex-determining decision.

Whether germ cells play an active or passive role during somatic cell differentiation of the mammalian gonad has been a long-standing question [11]–[13]. In XY gonads, loss of germ cells does not disrupt the ability of Sertoli cells to undergo morphological reorganization to form testis cords. However, germ cells are critical for morphological development of the ovary at birth, when clusters of germ cells break down and primordial follicles form, consisting of an oocyte surrounded by a single layer of pregranulosa cells [11], [13]–[15].

Whether germ cells also function to maintain the fate of the granulosa cell lineage, possibly by repressing aspects of Sertoli differentiation, is unknown. This proposition arose from the observation that a number of mutants that cause a loss of germ cells result in postnatal transdifferentiation of the granulosa cell lineage. In these cases, granulosa cells acquired morphological characteristics of Sertoli cells such as a tripartite nucleolus, basally located nuclei, and arrangement into cord-like structures [14] and transitioned from expressing ovarian markers, such as FOXL2, to expressing markers of Sertoli cells, such as SOX9 [16].

Some of the earliest cases of transdifferentiation were observed in female freemartin cattle where a loss of germ cells in response to hormones from the male co-twin was associated with the development of male cord-like structures in the ovary [17], [18]. In rodent models, exposure of ovaries to AMH [19], [20], double knockout of the two estrogen receptors (ERαβKO) [21], [22] and null mutations in the gene encoding P450 aromatase (*Cyp19a1*; ArKO) [22], [23] result in similar cord-like structures appearing in the postnatal ovary following germ cell loss. Mouse mutants for several genes expressed in the female supporting cell lineage, including *Wnt4* or *Rpso1* single mutants and the *Wnt4; Foxl2* double mutant, undergo a germ cell loss after meiotic entry and show evidence of sex reversal near birth [24]–[27]. However, not all cases of transdifferentiation can be attributed to germ cell loss. In the *Foxl2* mutant, postnatal transdifferentiation of granulosa cells occurred prior to oocyte loss [16], [28], indicating that the loss of germ cells was not responsible for the loss of granulosa cell fate.

To separate the effects of germ cell depletion from other somatic mutations, experimental manipulations that directly deplete ovaries of germ cells were performed, but these have also shown variable effects on ovarian differentiation. At postnatal stages, irradiation of rat ovaries did result in the appearance of testis cord-like structures [29]. While transdifferentiation induced by irradiation has not been reported in mouse, depletion of germ cells at different stages of postnatal ovarian development using Diptheria toxin did not lead to transdifferentiation [16]. Thus, the role of germ cells in establishing and maintaining ovarian fate after birth is still in question.

Depletion of primordial germ cells at the earliest stages of gonad development was previously performed using both chemical and genetic methods. Busulfan-induced germ cell depletion in rat embryos did not cause prenatal ovarian sex reversal based on histological examination [15]. Similarly, mutations of the white spotting locus (*Kit*) resulted in germ cell loss, but spermatogenic cords were not observed in the ovary before or after birth [30]. However, both of these studies were performed prior to the development of molecular markers of ovarian differentiation. While morphological changes reminiscent of Sertoli development were not observed, the possibility remained that the expression of genes that distinguish pregranulosa cells from Sertoli cells, such as FOXL2 and SOX9, might be altered. Such changes could potentially affect the ability of pregranulosa cells to form follicles at birth.

To establish a baseline for the major somatic subpopulations that exist in the fetal ovary, we first characterized the expression of a number of markers of somatic cell types common to the testis and ovary, as well as new markers for the ovary. We then examined the expression of these ovarian cell types in the context of early germ cell depletion using two different methods to ablate germ cells: chemical disruption using the chemotherapy drug busulfan and genetic disruption using the *Kit^Wv^* mutation [31]. Consistent with previous morphological studies, we found that the loss of germ cells did not impact the establishment or maintenance of multiple ovarian cell lineages including granulosa cells.

## Materials and Methods

### Mouse Strains and Genotyping

All animals were maintained and experiments were conducted according to the Institutional Animal Care and Use Committee of the Duke University Medical Center and NIH guidelines (Permit Number: A168-11-07). The *Wnt4-eGFPCre (Wnt4^GC/+^)* allele was generated using the same targeting scheme used for *Wnt4-eGFPCreER^T2^* mice [32]. *αSma-EYFP* mice (obtained from J. Lessard; [33]) were maintained on a mixed CD-1/FVB genetic background. *Oct4-GFP^Tg^* [34], *Wnt4^GC/+^* and *Kit^Wv/+^* (C57BL/6J-*Kit^W-v^*/J; Jackson Laboratory [31]) mice were maintained on a C57BL/6J genetic background.

For the *Wnt4^GC^* allele, Cre genotyping was used to distinguish wild type embryos from embryos carrying the mutant allele, and the gonad phenotype was used to distinguish heterozygous from homozygous mutants. The irregular development of the mesonephric ducts in both sexes, or the presence of vasculature in XX gonads, was characteristic of a homozygous mutant. For the *Kit^Wv^* mutation, a TaqMan SNP Genotyping Assay (Applied Biosystems) was developed and run on a StepOnePlus thermal cycler (Applied Biosystems) following the supplier's protocol. Primer and probe sequences (5′-3′) are as follows: Forward primer GCTACCTGGGCAATCACATGAATAT; Reverse primer TGAGTCTCGAGTTGCCATCTCT; FAM-conjugated probe for the *Kit^Wv^* allele CATGCATGGTGGGAG; and VIC-conjugated probe for the *Kit^+^* allele CATGCACGGTGGGAGG.

### Matings

To generate wild type embryos for immunofluorescent analysis, α*Sma-YFP^Tg/Tg^*, *Oct4-GFP^Tg/Tg^* and *Wnt4^GC/+^* males were crossed to CD-1 (Charles River) females in timed matings. For comparison of ovaries with and without germ cells, *Oct4-GFP^Tg/Tg^* males were crossed to CD-1 females, and pregnant females were injected intraperitoneally with 10–30 mg of busulfan (Sigma) dissolved in 50% DMSO, an equivalent volume of 50% DMSO, or left uninjected. No difference was observed between mock injected and uninjected mice, and they were used interchangeably as controls. *Kit^Wv/+^* heterozygous mice were intercrossed to generate *Kit^Wv/Wv^* embryos depleted of germ cells.

### Immunofluorescence

Following timed matings, gonads were dissected from embryos and fixed for several hours or overnight at 4°C in 4% paraformaldehyde. Samples were embedded in OCT and cryosectioned (18 µm) or whole mount immunostained with antibodies against the following markers: p27 (Santa Cruz, 1∶500), GFP (Aves lab, 1∶500 or Molecular Probes, 1∶1000), laminin (a kind gift from Harold Erickson; 1∶500), laminin (Neomarkers, 1∶500), MAFB (Bethyl Labs, 1∶2000), SOX9 (Millipore, 1∶2000), FOXL2 (Novus Biologicals, 1∶200 or a kind gift from Reiner Veitia, 1∶250), GATA4 (Santa Cruz, 1∶100), PECAM1 (BD BioScience, 1∶250), MIS/AMH (Santa Cruz, 1∶500), WT1 (Santa Cruz, 1∶100) and SCP3 (Novus Biologicals, 1∶500). Primary antibodies were detected by Cy2-, Cy3- and Cy5-conjugated secondary antibodies (Jackson ImmunoResearch Laboratories, 1∶500) or Alexa Fluor 488- and Alexa Fluor 647-conjugated secondary antibodies (Molecular Probes, 1∶500). Samples were mounted with 2.5% DABCO (Sigma-Aldrich) in 90% glycerol and imaged on a Leica SP2 confocal microscope.

## Results

### Characterization of the embryonic ovarian cell lineages

**Figure 1:**
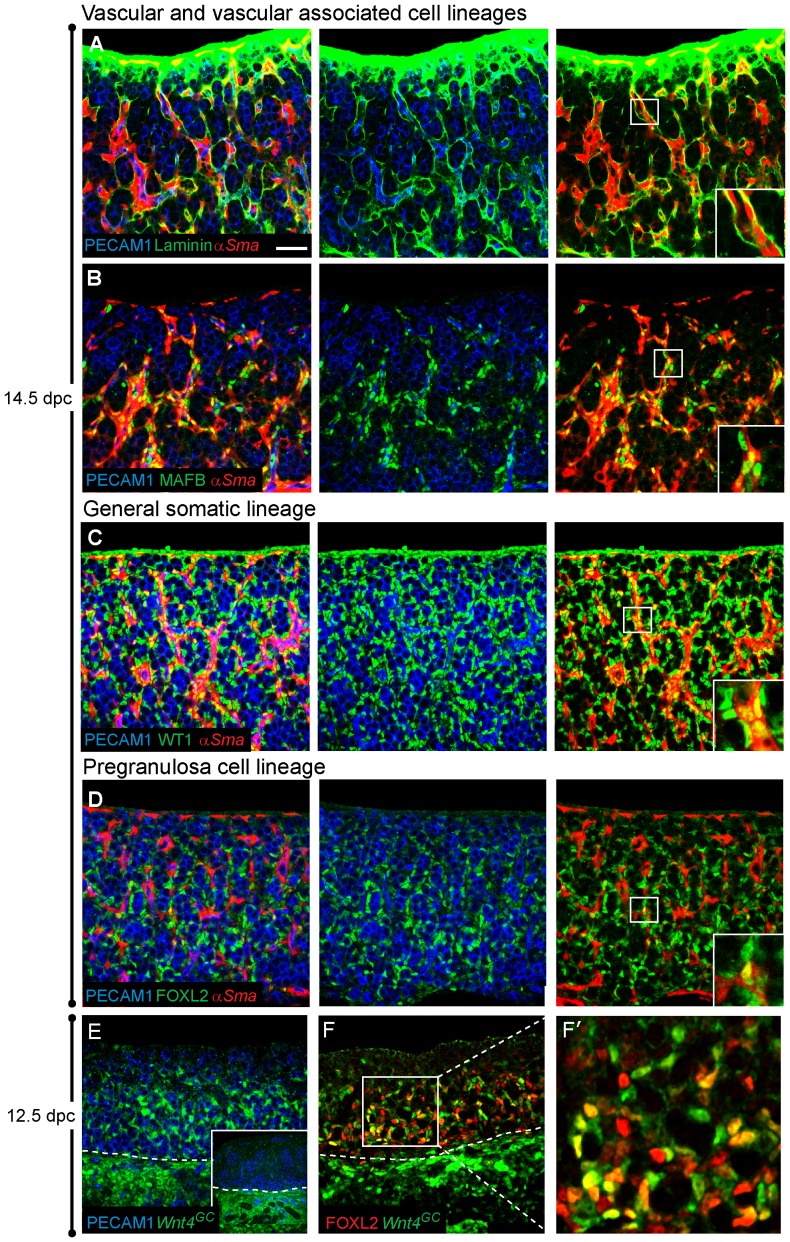
Characterization of ovarian cell lineages. (A–D) Ovaries were dissected from 14.5 dpc embryos expressing *αSma-EYFP* (red) and were immunostained for PECAM1 to label germ cells and vasculature (blue) and an additional somatic marker (green). (A) Laminin (green) is observed in the coelomic domain and around *αSma-EYFP*-expressing cells and vasculature (blue). (B) MAFB (green) marks both the nuclei of *αSma-EYFP-*expressing cells and non-*αSma-EYFP* expressing cells. Both cell types are closely associated with vasculature. (C) WT1 (green) is expressed in all non-endothelial somatic cells of the ovary, including *αSma-EYFP-*positive cells (C, yellow nuclei, inset in right panel). (D) FOXL2 (green), a marker of the pregranulosa cell lineage, is expressed in *αSma-EYFP-*negative somatic cells. *αSma-EYFP*/FOXL2 double-positive cells were occasionally observed (D, inset in right panel). (E–F) Gonads were collected from 12.5 dpc *Wnt4^GC^* embryos. *Wnt4*-expressing cells, only detectable using an antibody to GFP (green), were present throughout the mesonephros of both XX (E) and XY samples (inset in E). *Wnt4*-expressing cells were present in the gonad only in XX samples (E) and *Wnt4* expression overlapped with FOXL2 in many cells (F and F′). Whole mount immunostaining was performed on all samples. The scale bar in panel (A) represents 50 µm in panels A–F, 18.5 µm in all insets except E (20.8 µm) and 16.5 µm in panel G′.

In contrast to the distinct structural organization of the testis, the embryonic ovary undergoes few obvious morphological changes. At 12.5 dpc, when testis cords are first observed in XY gonads, XX gonads appear as a heterogeneous mixture of somatic and germ cells. By 14.5 dpc, a subtle organization is apparent upon histological or immunological examination, where meiotic germ cells are arranged into clusters referred to as ovigerous cords [35]. Near birth, these clusters break down and primordial follicles form, a process that is dependent on the presence of germ cells [36]. To determine if germ cells influence the establishment or maintenance of the major ovarian cell lineages, we first identified markers and characterized the different cell types present in the ovary using confocal immunofluorescence. Figure 1 and Figure S1 summarize the main somatic cell types distinguished in the embryonic ovary between 12.5–14.5 dpc: (1) vascular endothelial cells, (2) vasculature-associated cells, (3) cells residing in or near the coelomic epithelium and (4) pregranulosa cells.

In males, endothelial cells that reside in the gonad-mesonephros border are recruited into the gonad to form a well-defined vasculature and coelomic vessel [33], [37], [38] that demarcates and instructs formation of the testis cords [39], [40]. Outside of testis cords, in close proximity to the vasculature, resides a diverse population of interstitial cells, some of which express members of the MAF transcription factor family and give rise to the steroid-producing fetal Leydig cells [4]. The functional significance of these parallel cell types in the ovary is currently unclear. Similar to the testis, the embryonic ovary contains vascular endothelial cells surrounded by proximate cells that express MAFB alone or co-express MAFB and *α-smooth muscle actin-EYFP* (α*Sma-EYFP*; Figure 1B). We found that pockets of germ cells are surrounded by pregranulosa cells, and broken into clusters by lines of endothelial cells and other vascular associated cells outside the laminin surrounding the ovigerous cord structures (Figure 1A).

Several transcription factors essential for the early formation of the gonad in both sexes include WT1, GATA4 and SF1 [41]–[43]. At 14.5 dpc, WT1 is expressed in all somatic cells of the ovary, except endothelial cells (Figure 1C). This was also seen for SF1 and GATA4 at both earlier (12.5–13.5 dpc) and later (17.5 dpc) stages (Figure S1 and data not shown). While it was previously reported that GATA4 and SF1 expression levels are downregulated after 13.5 dpc [44], [45], we found persistent GATA4 and SF1 expression in the ovary at the stages we examined. This may reflect differences in antibody or detection sensitivities.

A subset of somatic cells, the supporting cells, surrounded nests of germ cells, retained expression of these transcription factors and also expressed FOXL2 (Figure 1D). These cells are referred to as supporting cells because they are closely associated with germ cells throughout development and give rise to granulosa cells in the first wave of growing follicles that form after birth [3]. FOXL2-positive cells were initially observed close to the mesonephros and gradually extended out towards the coelomic surface, filling the future medullary region of the ovary but excluded from a region near and including the coelomic surface that expressed either WT1 alone or was α*Sma-EYFP*/WT1-double positive (Figure S1, also [3]). Near birth, FOXL2-positive cells were located in both the medulla and cortex of the ovary, though still excluded from the coelomic surface. FOXL2-expressing cells were usually distinct from the α*Sma-EYFP*/MAFB population; however, a few cells expressing both α*Sma-EYFP* and FOXL2 were detected at both early and late stages (inset in Figure 1D).

Based on previous *in situ* and microarray data, *Wnt4* was found to be expressed in both male and female gonads before 11.5 dpc, subsequently becoming female specific [27], [46]. To investigate which cell type expresses *Wnt4* in the ovary, we used a *Wnt4-eGFPCre* (*Wnt4^GC^*) targeted allele as a transcriptional reporter. *Wnt4^GC^* was detected in the mesonephros of XX and XY gonads at 12.5 dpc but was specific to somatic cells of the XX gonad (Figure 1E; XY gonad shown in inset). Co-labeling *Wnt4^GC^* gonads with FOXL2 revealed that *Wnt4* was also expressed in the pregranulosa cell lineage of the ovary, closely overlapping FOXL2 expression. Due to variability in the brightness of each marker, we could not determine whether some cells express either *Wnt4^GC^* or FOXL2 alone. Similar to FOXL2, *Wnt4* is not expressed in the cells closest to the coelomic surface. Expression of the reporter was very weak after 14.5 dpc and could not be detected at 17.5 dpc (data not shown).

In summary, gonadal somatic markers were expressed in non-endothelial cells. A subset of these cells expressed MAFB and α*Sma-EYFP* (vascular associated), while another subset, the pregranulosa cell lineage, expressed FOXL2 and *Wnt4* (supporting cells). Consistent with this result, *Wnt4* expression was detected specifically in the supporting cell lineage of the XX gonad by microarray analysis of individual cell lineages from the early ovary and testis [47], [48].

### The effect of germ cell loss on ovarian differentiation

**Figure 2:**
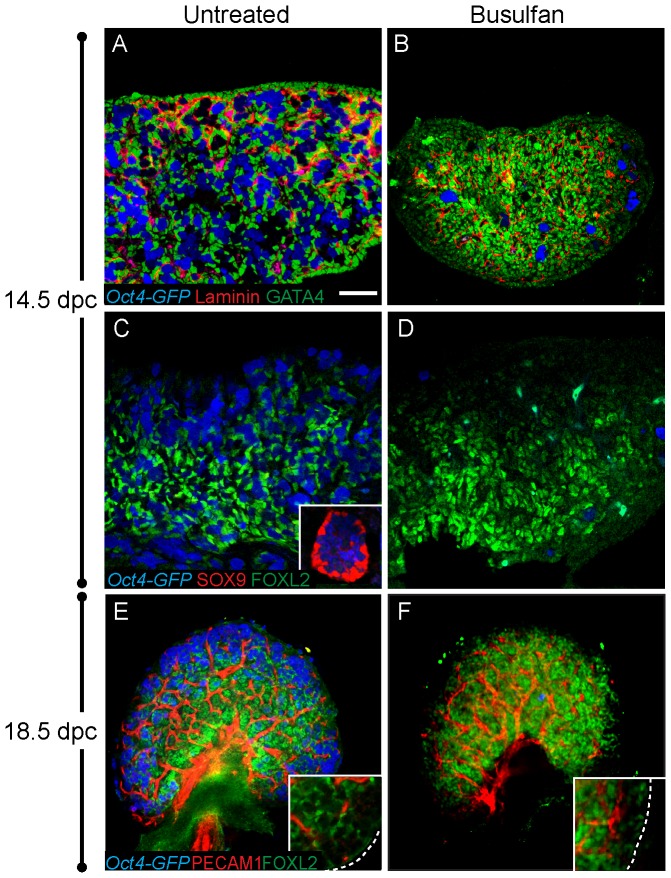
Loss of germ cells does not affect expression of ovarian markers. *Oct4-GFP* transgenic mice were injected with busulfan at 10.5 or 11.5 dpc and gonads were dissected at 14.5 or 18.5 dpc, respectively. *Oct4-GFP*-expressing germ cells (blue) were severely depleted, or absent, from treated samples (B, D, and F). (A–B) At 14.5, GATA4 (green) was expressed in all somatic cells of control and germ-cell depleted samples and laminin (red) expression was not detectably altered. (C–D) Control and busulfan treated gonads at 14.5 dpc were immunostained for SOX9 (red) and FOXL2 (green). FOXL2 was similarly expressed and no SOX9 was detected in control or treated XX gonads. An inset in (C) shows SOX9 expression (and no FOXL2) in an XY gonad at the same stage (the exposure levels for SOX9 and FOXL2 were identical for all samples in panels C and D). (E–F) The distribution of FOXL2 (green) and vasculature (red) was similar in control and busulfan-treated ovaries at 18.5 dpc (insets show an enlarged view of the cortex). Samples in A–D were cryosectioned then immunostained. Whole mount immunostaining was performed on samples in E–F. The scale bar in panel (A) represents 50 µm in panels A–D (including the inset in C), 100 µm in panels E–F and 24.5 µm in insets within E–F.

Several mutations result in both the loss of germ cells and postnatal sex reversal. Because sex reversal is observed after germ cell loss in these cases, it has been proposed that germ cells are required to establish or maintain the ovarian cell fate (for review see [11], [14]). To test this hypothesis, we investigated the effect of germ cell depletion on the establishment of the major ovarian cell lineages during fetal life. This was accomplished by treating pregnant females with busulfan to deplete germ cells at a stage when they are just arriving in the genital ridge, between 10.5–11.5 dpc. We used *Oct4-Gfp* transgenic mice [34] so that the presence of germ cells could be easily determined. Mice injected at 10.5 dpc were severely depleted of germ cells by 12.5 dpc. At 14.5 dpc, GATA4, which marks all non-endothelial ovarian somatic cells, was expressed normally in germ cell-depleted ovaries and laminin deposition surrounded clusters of somatic cells in the absence of germ cells (Figure 2A, B). The pregranulosa cell lineage, as marked by FOXL2 expression, was established normally and a marker of Sertoli cells, SOX9, was not detected (Figure 2C, D; inset in 2C shows positive staining for SOX9 expression in a control testis). Mice injected with busulfan at 11.5 dpc were analyzed at 17.5–18.5 dpc, just before birth. Although treated ovaries were markedly smaller, somatic markers were expressed similar to controls, and there was no evidence of sex reversal (Figure 2E, F). Additionally, no defect was observed in formation of the vasculature (marked by PECAM1) or the number of vascular associated cells (marked by Laminin) (Figure 2B, F).

**Figure 3:**
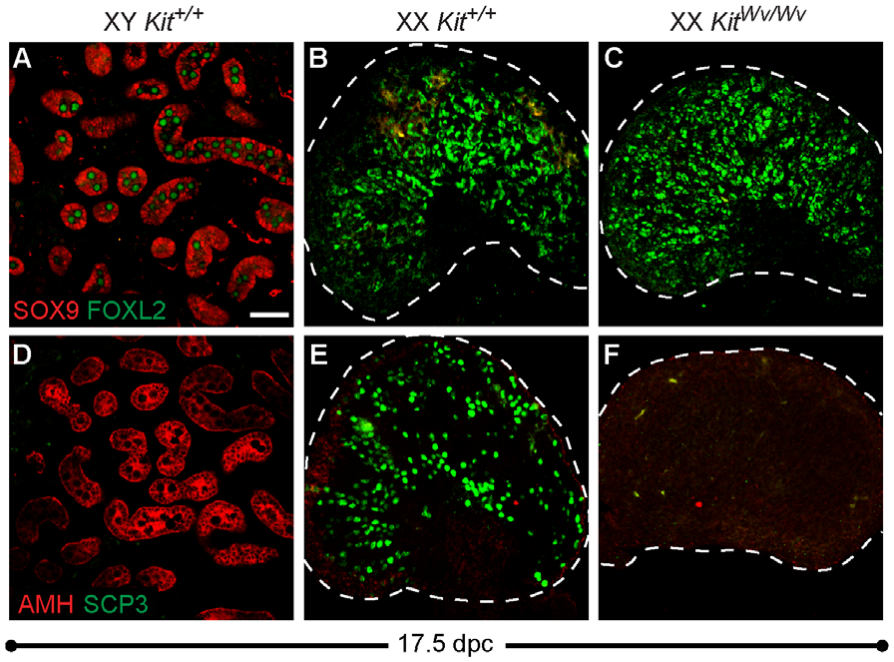
Ovaries lacking germ cells do not transdifferentiate towards a Sertoli cell fate. (A–C) SOX9 (red) and FOXL2 (green) immunostaining of cryosectioned 17.5 dpc gonads. (A) XY *Kit^+/+^* testes express SOX9, but not FOXL2 (XY germ cells exhibit high background staining). (B–C) XX *Kit^+/+^* and *Kit^Wv/Wv^* ovaries express only FOXL2 and show no evidence of transdifferentiation. (D–F) AMH (red) and SCP3 (green) immunostaining of the same samples as in A–C. (D) As expected, XY *Kit^+/+^* testes express AMH, but not the meiotic germ cell marker SCP3. (E–F) XX *Kit^+/+^* and *Kit^Wv/Wv^* ovaries do not express AMH. SCP3 immunostaining confirms significant germ cell loss in *Kit^Wv/Wv^* ovaries (E,F). All samples were cryosectioned then immunostained. The scale bar represents 50 µm in all panels. The dotted line demarcates the boundary of the ovary.

To examine the effects of germ cell loss using a non-chemically based method, we next analyzed *Kit^Wv/Wv^* mutants, which show a severe loss of germ cells during the migratory phase [31]. To determine if germ cell loss caused transdifferentiation of pregranulosa cells near birth, the expression of two Sertoli cell markers, SOX9 and AMH, were examined in XX *Kit^+/+^* and XX *Kit^Wv/Wv^* ovaries. As expected, SOX9 and AMH are expressed in XY *Kit^+/+^* Sertoli cells at 17.5 dpc. Cells in XY gonads do not express the pregranulosa cell marker FOXL2 or the meiotic germ cell marker SCP3 (Figure 3A, B). SCP3-positive germ cells are widespread in XX *Kit^+/+^* ovaries but were severely depleted in *Kit^Wv/Wv^* samples, confirming a near complete loss of germ cells in the homozygous mutants (Figure 3E, F). However, consistent with the results of the busulfan experiments, we observed no difference in FOXL2 expression between *Kit^+/+^* and *Kit^Wv/Wv^* samples, nor did we observe any evidence of transdifferentiation towards a Sertoli cell fate (Figure 3B, C, E, F). Taken together, these results suggest that loss of germ cells alone does not result in morphological evidence of sex reversal or disrupt the prenatal establishment or maintenance of the ovarian cell types, at least as defined by the markers we investigated.

## Discussion

### Germ cells are not required for ovarian development during embryogenesis

In mammals, the role of XX germ cells as potential regulators of ovarian development has been extensively questioned (reviewed in [11], [14]). In the testis, germ cell depletion does not disrupt the formation of testis cords; however, near birth, germ cells are absolutely required for the formation of ovarian follicles [11], [49]. The finding that certain scenarios cause a loss of germ cells prior to transdifferentiation of the granulosa cell lineage, led to the idea that germ cells may play an active role in repressing the male pathway.

Previous studies analyzed busulfan-treated fetal rat ovaries, as well as mouse *Kit^W/Wv^* ovaries, and relying on ultrastructural morphological analyses, came to the conclusion that germ cells are not required for the differentiation of the somatic lineages during embryonic stages [15], [30]. Here we used molecular markers to examine the commitment and maintenance of somatic cell fate in germ cell-depleted ovaries. Using a variety of markers for different cell types, we characterized the four predominant somatic cell lineages in the ovary: (1) Vascular endothelial cells (PECAM1-positive), (2) Vasculature associated cells (α*Sma-EYFP*- and MAFB-positive), (3) General somatic cells (GATA4-, SF1- and WT1-positive) and (4) granulosa cell precursors (*Wnt4-* and FOXL2-positive). An examination of these lineages after busulfan treatment revealed no obvious defects in their establishment or maintenance. Using a second model of germ cell depletion, the *Kit^Wv^* mutation, we also found no evidence of disrupted ovarian development or transdifferentiation towards the Sertoli cell fate. In the absence of a detectable defect in the somatic ovarian program, we conclude that the subsequent failure to form follicles at perinatal stages is attributable to the absence of germ cells; however, we cannot rule out the possibility that defects arise in somatic cells at birth.

Consistent with prior experiments [15], [30], our results support the conclusion that depletion of germ cells at pre-meiotic stages does not lead to sex reversal. Some evidence suggests that the influence of germ cells over the ovarian somatic environment, and the ability to repress various aspects of testis development, may vary according to developmental stage [14]. We previously found disruptions in endothelial cell migration and testis cord formation when XY gonadal cells were exposed to meiotic germ cells, but not pre-meiotic germ cells or XX gonads that were depleted of germ cells [50], suggesting that meiotic germ cells, not pre-meiotic, may function in repression of the testis pathway. The majority of transdifferentiation cases occur when germ cells survive the pre-meiotic stage of germ cell development, and die following meiotic entry. This is the case for the *Rspo1*, *Wnt4* and *Wnt4; Foxl2* mutants that undergo isolated perinatal sex reversal [24]–[27] and the AMH transgenic that exhibits transdifferentiation during postnatal stages [19]. Germ cell depletion just after birth, or at late postnatal stages, leads to transdifferentiation in some cases (irradiation of rat ovaries; [29]), but not others (*Fig1a*, *Diptheria Toxin*; [16], [51]). Importantly, transdifferentiation can occur in the presence of germ cells, as FOXL2-deficient granulosa cells were shown to transdifferentiate prior to oocyte loss [16], [28].

Our experiments, which removed germ cells at the pre-meiotic stage, analyzed the role of germ cells in establishing and maintaining the ovarian cell fate during fetal stages of development. The results support the conclusion that a loss of germ cells at this early stage does not alter the fate of somatic cells in the fetal ovary. However, one possibility is that granulosa cells acquire the competence for transdifferentiation following contact with meiotic germ cells. If germ cells are lost prior to meiotic entry, pregranulosa cells do not proceed through this differentiation step and cannot transdifferentiate. This would imply that pregranulosa cell-germ cell interactions, which are critical after birth for folliculogenesis, also play a role during fetal ovarian stages. Further studies that specifically deplete meiotic germ cells at prenatal stages, in the absence of a genetic mutation in the soma, would clarify whether meiotic germ cell loss has detrimental effects on granulosa cell differentiation and repression of Sertoli markers.

## Supporting Information

Figure S1 **Characterization of ovarian cell lineages from 12.5–13.5 dpc.** (A–F) Ovaries were dissected from 12.5 dpc (left panel) and 13.5 dpc embryos (right panel) and immunostained for PECAM1 to label germ cells and vasculature (blue), FOXL2 (green) and an additional somatic marker (red). (A) αSMA, (B) Laminin, (C) GATA4, (D) SF1, (E) WT1 and (F) LHX9. Whole mount immunostaining was performed on all samples. Scale bar in (A) represents 50 µm in all panels.
(TIF)
