# Supplementary figures and images for: Germ Cells Are Not Required to Establish the Female Pathway in Mouse Fetal Gonads
Source: PLoS One. 2012 Oct 16;7(10):e47238. doi: 10.1371/journal.pone.0047238 (PMC3473035; doi:10.1371/journal.pone.0047238)

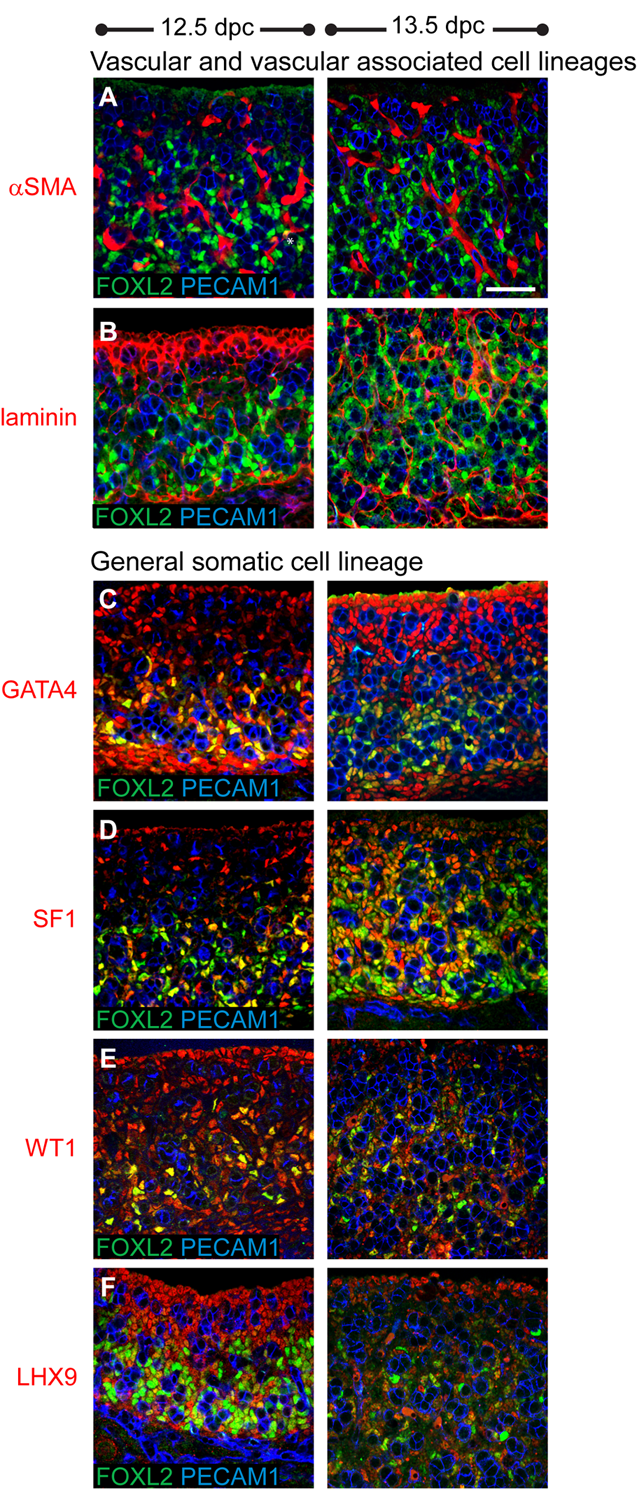

Supplement: Figure S1 — Characterization of ovarian cell lineages from 12.5–13.5 dpc. (A–F) Ovaries were dissected from 12.5 dpc (left panel) and 13.5 dpc embryos (right panel) and immunostained for PECAM1 to label germ cells and vasculature (blue), FOXL2 (green) and an additional somatic marker (red). (A) αSMA, (B) Laminin, (C) GATA4, (D) SF1, (E) WT1 and (F) LHX9. Whole mount immunostaining was performed on all samples. Scale bar in (A) represents 50 µm in all panels. (TIF) [file pone.0047238.s001.tif]
